# A repeated cross-sectional analysis on the economic impact of SARS-CoV-2 pandemic at the hospital level in Italy

**DOI:** 10.1038/s41598-023-39592-7

**Published:** 2023-07-31

**Authors:** Filippo Trentini, Oriana Ciani, Elena Vanni, Simone Ghislandi, Aleksandra Torbica, Elena Azzolini, Alessia Melegaro

**Affiliations:** 1 Dondena Centre for Research on Social Dynamics and Public Policy, Bocconi University, 1 Roentgen St., Milan, Italy; 2 Covid Crisis Lab, Bocconi University, Milan, Italy; 3 Center for Health Emergencies, Bruno Kessler Foundation, Trento, Italy; 4 Centre for Research on Health and Social Care Management (CERGAS), SDA Bocconi School of Management, Milan, Italy; 5 IRCCS Humanitas Research Hospital, Milan, Italy; 6 Department of Social and Political Sciences, Bocconi University, Milan, Italy; 7 Department of Biomedical Sciences, Humanitas University, Milan, Italy

**Keywords:** Infectious diseases, Respiratory tract diseases, Health care economics, Health policy, Health services, Public health

## Abstract

Italy was the first country in Europe to be hit by the Severe Acute Respiratory Syndrome Coronavirus 2. Little research has been conducted to understand the economic impact of providing care for SARS-CoV-2 patients during the pandemic. Our study aims to quantify the incremental healthcare costs for hospitalizations associated to being discharged before or after the first SARS-CoV-2 case was notified in Italy, and to a positive or negative SARS-CoV-2 notified infection. We used data on hospitalizations for 9 different diagnosis related groups at a large Italian Research Hospital with discharge date between 1st January, 2018 and 31st December 2021. The median overall costs for a hospitalization increased from 2410EUR (IQR: 1588–3828) before the start of the pandemic, to 2645EUR (IQR: 1885–4028) and 3834EUR (IQR: 2463–6413) during the pandemic, respectively for patients SARS-CoV-2 negative and positive patients. Interestingly, according to results of a generalized linear model, the highest increases in the average costs sustained for SARS-CoV-2 positive patients with respect to patients discharged before the pandemic was found among those with diagnoses unrelated to COVID-19, i.e. kidney and urinary tract infections with CC (59.71%), intracranial hemorrhage or cerebral infarction (53.33), and pulmonary edema and respiratory failure (47.47%). Our study highlights the economic burden during the COVID-19 pandemic on the hospital system in Italy based on individual patient data. These results contribute to the to the debate around the efficiency of the healthcare services provision during a pandemic.

## Introduction

Italy was the first country in Europe to be hit by the Severe Acute Respiratory Syndrome Coronavirus 2 (SARS-CoV-2). To date, Lombardy remains the worst affected region in the country, with a total of > 4.1 million confirmed cases of Coronavirus Disease 19 (COVID-19) and > 46.000 deaths reported since the start of the pandemic up to June 2023. Almost a third of confirmed SARS-CoV-2 cases occurred in Lombardy’s capital, Milan. The emergence of the pandemic exerted an enormous pressure on the Italian healthcare system leading to a 22% reduction in its capacity of hospital care compared to the averaged in the previous three years^1^ and a significant increase in excess mortality associated with the COVID-19 pandemic^2,3^.

Several public health measures were enforced to mitigate the effect of the pandemic, whilst within hospitals and hospital networks a number of actions were put in place to cope with (i) care of an increasing number of acutely ill patients, and (ii) the need to isolate cases and prevent infection of patients negative at admission. These went from increase of ICU beds and acquisition of related devices and consumables to reallocation of spaces and resources, testing policies and use of personal protective equipment^4^.

Several studies highlighted the substantial change in patterns of hospital admission during the pandemic and showed reduced admission rates across a heterogeneous range of diagnoses especially during the first half of 2020^5–7^. The impact of containment and mitigation measures resulted in a diagnostic delay for several conditions, and consequently a surge in relatively more severe cases requiring hospital care^8^. Other studies analyzed patterns of ICU admission and suggested that limited health-care resources during 2020 forced a shift in admission criteria to prioritize patients with higher chances of survival^9^.

While a large amount of Italian studies focused on pathogen transmission, disease epidemiology and the pressure on hospitals^9–11^, little research has been conducted to understand the economic impact at the hospital level of providing care for SARS-CoV-2 positive^12–15^ and compare it with a pre-pandemic period.

Our study aims to investigate the full and incremental economic costs of hospitalizations during the COVID-19 pandemic in a repeated cross-sectional analysis framework. More specifically, we aim to quantify the incremental costs including diagnostics, medical devices, critical care provision, personnel for each episode of hospitalization associated to being discharged after the first case of SARS-CoV-2 was notified in Italy with or without a SARS-CoV-2 positive test for eight different Diagnosis Related Groups (DRGs) with respect to a pre-pandemic baseline.

## Results

Table 1 shows the characteristics of the 6396 hospitalization episodes analyzed. Overall, 2191 (34.3%) hospitalizations with discharge date before 21st February 2020 and 4205 (65.7%) after. Of the latter, 2347 (55.8%) were SARS-CoV-2 positive (vs. 1858 negative).

**Table 1:** Characteristics of hospitalization by discharge period and SARS-CoV-2 status.

Variable		Discharge before 21/02/2020 (pre-pandemic)		Discharge after 21/02/2020 (pandemic) SARS-CoV-2 negative		Discharge after 21/02/2020 (pandemic) SARS-CoV-2 positive	
		N (%)	MEDIAN (IQR)	N (%)	MEDIAN (IQR)	N (%)	MEDIAN (IQR)
Age		2191 (100)	75 (65–80)	1858 (100)	75 (65–80)	2347 (100)	70 (55–80)
Male patients		1255 (57.3)	–	1041 (56.0)	–	1457 (62.1)	–
Length of stay	Total (days)	2191 (100)	9 (6–15)	1858 (100)	9 (6–14)	2347 (100)	11 (7–19)
	Surgery (hours)	79 (3.6)	0.9 (0.7–1.3)	58 (3.1)	0.9 (0.6–1.1)	19 (0.8)	0.9 (0.7—1.4)
	ICU (days)	142 (6.5)	4.5 (2.0–9.0)	76 (4.1)	3.5 (1.8–9.3)	91 (3.9)	10.0 (6.0–17.0)
	UCC (hours)	17 (0.8)	1.6 (0.5–3.2)	1 (0.1)	0.9 (0.9–0.9)	14 (0.6)	3.4 (1.0–6.3)
Type of admission	Elective	46 (2.1)	–	73 (3.9)	–	156 (6.6)	–
	Emergency room	2145 (97.9)	–	1785 (96.1)	–	2191 (93.4)	–
DRG CODE	14	497 (22.7)	–	523 (28.1)	–	33 (1.4)	–
	79	252 (11.5)	–	211 (11.4)	–	897 (38.2)	–
	80	60 (2.7)	–	50 (2.7)	–	170 (7.2)	–
	87	241 (11)	–	126 (6.8)	–	441 (18.8)	–
	89	647 (29.5)	–	519 (27.9)	–	415 (17.7)	–
	90	244 (11.1)	–	143 (7.7)	–	168 (7.2)	–
	320	178 (8.1)	–	247 (13.3)	–	19 (0.8)	–
	565	31 (1.4)	–	23 (1.2)	–	157 (6.7)	–
	566	41 (1.9)	–	16 (0.9)	–	47 (2.0)	–

Patients who tested positive for SARS-CoV-2 were slightly younger than those who tested negative and those discharged before the beginning of the pandemic in Italy (median age 70 vs. 75). The percentage of male patients among those who tested positive for SARS-CoV-2 was higher (62.08%) than the percentage of male patients among those who tested negative (56.03%) and those discharged before the beginning of the pandemic in Italy (57.28%). The percentage of elective admissions slightly increased for hospitalization episodes during the pandemic, respectively 6.6% for those with a SARS-CoV-2 diagnosis and 3.9% for those without, with respect to before (2.1%).

Overall 156 (2.4%), 309 (4.8%) and 30 (0.5%) hospitalizations required respectively surgery, admission to an ICU or admission to an UCC. The rate of hospitalized patients admitted to ICU decreased from 6.5% before the pandemic to around 4% in patients discharged during the pandemic, and the median length of stay in ICU reached a peak of 10 days (IQR: 6–17) among patients who tested positive for SARS-CoV-2.

An exploratory analysis of the 10 most relevant principal diagnoses among the three groups (pre-pandemic, pandemic and SARS-CoV-2 negative, pandemic and SARS-CoV-2 positive) suggested there was not a big shift in the three groups except for the presence of Covid-19 related diagnoses in the group of positive inpatients discharged during the pandemic.

**Figure 1:**
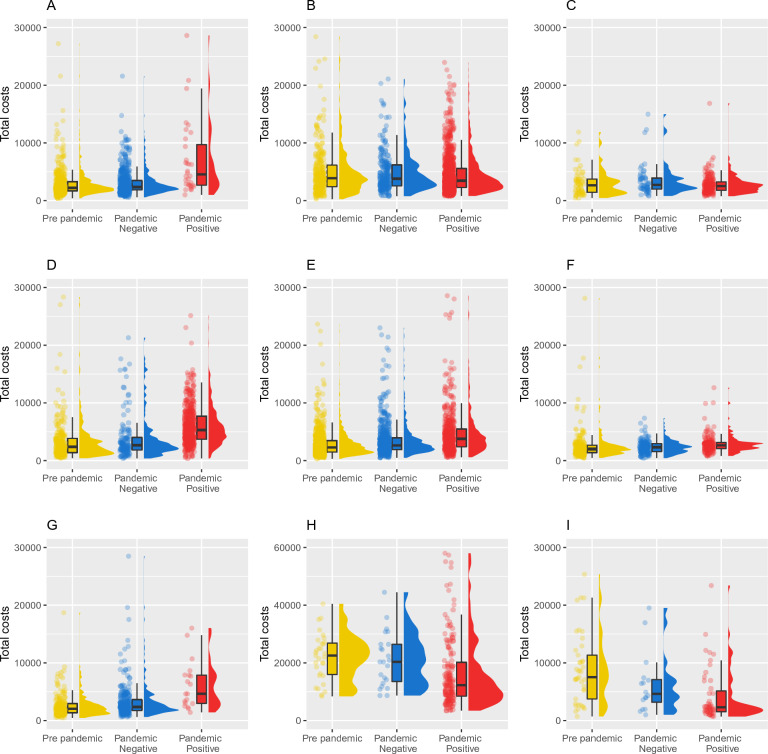
Distribution of costs (in EUR). Distribution of sustained cost for patients hospitalized for intracranial hemorrhage or cerebral infarction (014) (**A**), respiratory infections and inflammations with (079) (**B**) or without (080) (**C**) CC, pulmonary edema and respiratory failure (087) (**D**), simple pneumonia and pleurisy with (089) (**E**) and without (090) (**F**) CC, kidney and urinary tract infections with CC (320) (**G**), respiratory system diagnosis with ventilator support >  = 96 (565) (**H**) or < 96 h (566) (**I**), discharged in the pre-pandemic period (yellow), during the pandemic without a diagnosis for SARS-COV-2 (blue), and during the pandemic with a diagnosis for SARS-COV-2 (red).

**Figure 2:**
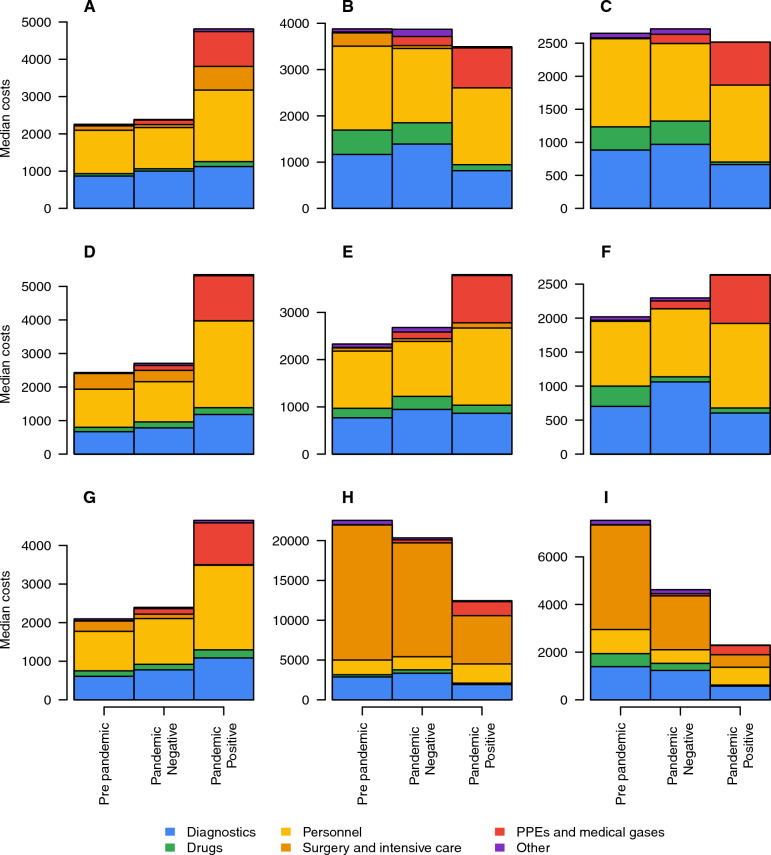
Stratification of costs. Median total costs stratified by different types of resources used for patients hospitalized for intracranial hemorrhage or cerebral infarction (**A**), respiratory infections and inflammations with (**B**) or without (**C**) CC, pulmonary edema and respiratory failure (**D**), simple pneumonia and pleurisy with (**E**) and without (**F**) CC, kidney and urinary tract infections with CC (**G**), respiratory system diagnosis with ventilator support >  = 96 (**H**) or < 96 h (**I**).

The median overall costs for a hospitalization were 2410 EUR (IQR: 1588–3828) before the start of the pandemic, 2645 EUR (IQR: 1885–4028) for patients discharged during the pandemic who tested negative and 3834 EUR (IQR: 2463–6413) for patients who tested positive for SARS-CoV-2. As shown in Figs. 1, 2 and Table S1 in the Appendix, costs for SARS-CoV-2 positive patients hospitalized and discharged during the pandemic were approximately twice as high as for negative patients for a series of conditions (i.e., 014—intracranial hemorrhage or cerebral infarction, 320—kidney and urinary tract infections with CC and 087—pulmonary edema and respiratory failure).

To compare the whole distribution of costs, we conducted a one-sided Kolmogorov Smirnov test to verify the hypothesis of equal cumulative distribution functions (CDFs) of costs versus the alternative that the CDF of hospitalization costs for patients with a positive SARS-CoV-2 diagnosis is lower, and these functions are shown in Fig. S1 of the Appendix. For certain conditions (i.e., 014—intracranial hemorrhage or cerebral infarction, 320—kidney and urinary tract infections with CC, 087—pulmonary edema and respiratory failure, 089—simple pneumonia and pleurisy with CC, and 090—simple pneumonia and pleurisy without CC) costs for SARS-CoV-2 positive patients were higher than costs for SARS-CoV-2 negative patients discharged during the pandemic, and for those who were discharged before the pandemic started (one-sided KS test *p* values < 0.001). Figure S1 in the Appendix shows the cumulative distributions of costs across different groups of patients and DRGs.

By stratifying the costs by type of resource consumed, we observed that among patients with respiratory infections (Fig. 2B and C and Table S1 in the Appendix), resources required for drugs and diagnostics slightly decrease for those who were discharged during the pandemic with a SARS-CoV-2 diagnosis. On the other hand, among patients with a diagnosis of intracranial hemorrhage or cerebral infarction, pulmonary edema and respiratory failure, simple pneumonia and pleurisy with and without CC and kidney and urinary tract infections with CC higher costs are mainly ascribable to an increase in costs for diagnostics, for personnel and for consumables like PPEs and medical gases, as shown in Figs. 2A, D–G and Table S1 of the Appendix. As highlighted by Fig. 2A–F and Table S1 of the Appendix, across all DRGS, the increase in hospitalization costs among SARS-CoV-2 negative patients discharged during the pandemic with respect those discharged before February 2020 is mainly due to an increase in costs for diagnostics and for consumables like PPEs and medical gases.

**Figure 3:**
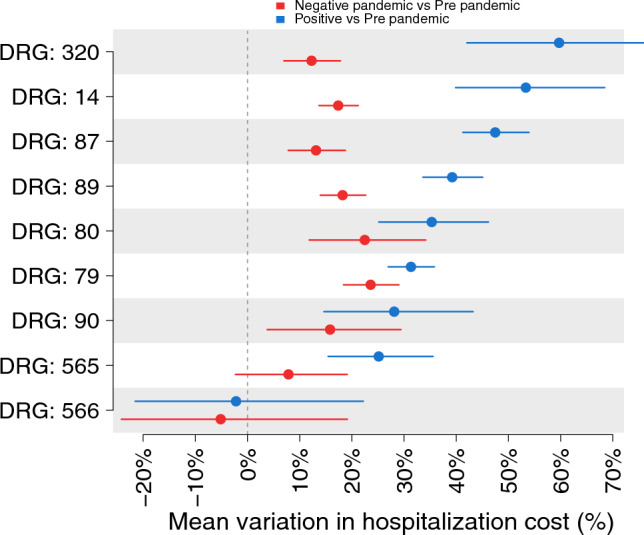
Results of the generalized linear model on the total costs. Average percentage increase/reduction of hospitalization costs for patients discharged during the pandemic with a positive or negative SARS-CoV-2 diagnosis with respect to patients discharged before the pandemic. Let ${\beta }_{group}$ denote the coefficient relative to the variable defining patients subgroups, such variations are obtained as $100*\left( {\exp^{{\beta_{group} }} } \right)$.

Figure 3 shows the results of the generalized linear models, which is presented as the baseline analysis since it was more conservative in terms of uncertainty of the estimates. The comparison between patients discharged during the pandemic with a SARS-CoV-2 positive test and those discharged before shows the highest increases in the average costs sustained for SARS-CoV-2 positive patients among those with a diagnosis of kidney and urinary tract infections with CC (59.71%, 95%CI: 42.03–80.16, *p* value < 0.001), intracranial hemorrhage or cerebral infarction (53.33%, 95%CI: 39.87–68.46, *p* value < 0.001), and pulmonary edema and respiratory failure (47.47%, 95%CI: 41.26–53.94, *p* value < 0.001). Among patients with a diagnosis of simple pneumonia and pleurisy with and without CC, the average increases are respectively 39.22% (95%CI: 33.61–45.08, *p* value < 0.001) and 28.11% (95%CI: 14.65–43.22, *p* value < 0.001), among those with respiratory infections and inflammation with and without CC, the average increases are respectively 31.32% (95%CI: 26.95–35.82, *p* value < 0.001) and 35.3% (95%CI: 25.15–46.17, *p* value < 0.001).

The lowest increases are found for patients with a diagnosis of respiratory system diagnosis with ventilator support >  = 96 and < 96 h, respectively 25.14% (95%CI: 15.45–35.56, *p* value < 0.001) and − 2.20% (95%CI: − 21.54–22.19, *p* value 0.84).

The average costs sustained for patients discharged during the pandemic with a SARS-CoV-2 negative test are found to be significantly higher than for those discharged before, except for those with a respiratory system diagnosis who required ventilator support. For all other patients, the increase in costs sustained for negative patients discharged during the pandemic varies between 12.27% (95%CI: 6.97–17.81, *p* value < 0.001) and 23.59% (95%CI: 18.39–29.02, *p* value < 0.001), respectively for patients with kidney and urinary tract infections with CC and for those with respiratory infections and inflammations without CC.

A check on any temporal trend over the four year time period on all pre-pandemic and negative patients was performed revealing no evidence of such effect, as shown in Fig. S2 of the Appendix. All model estimates are reported in Table S2 of the Appendix, along with *p* values, sample size and goodness of fit measures. Estimated increases in costs obtained by adopting an alternative model, i.e. a log-linear model, are in line with those obtained with the generalized linear model with absolute differences in the estimated increases of at most 9%, as shown in Fig. S3 and Table S2. Residual plots for both models are shown in Figs. S4 and S5 of the Appendix.

## Discussion

Our study, for the very first time, sheds light on the resources needed to take care of hospitalized cases during the COVID-19 pandemic in Italy. Our results suggest a median direct hospitalization cost between 2300 EUR and 12,450 EUR for a SARS-CoV-2 related admission, depending on the DRG. Previous work has estimated a median direct medical cost of 18,579 USD (17,524–19,609) for a person with symptomatic infection requiring hospitalization in the US^16^.

Our results suggest that the pandemic had significant impact on the direct healthcare costs in the hospital setting: the median hospital costs increased significantly with the SARS-CoV-2 infection across all DRGs (from + 621.0 EUR in DRG 090 to + 2917.5 EUR in DRG 087). These findings were confirmed in multivariate analysis, accounting for all available confounders, with an increase in hospital costs associated with SARS-CoV-2 infection with respect to pre pandemic hospitalizations between 25.04% (DRG 565) and 54.7% (DRG 087). When compared to the DRG tariffs add-on for the emergency period of 3713 EUR for each discharge without ICU admission, and 9617 EUR for each discharge with at least one day in the ICU deliberated by the Italian Ministry of Health, these increments seem to be well compensated (Decree 12/8/2021).

Results have also been disentangled by cost source and type of intervention. The increase in the costs following the pandemic is confirmed across all the presented dimensions, with some exception. In particular, for many DRGs, SARS-CoV-2 negative patients in the pandemic periods were allocated less resources for personnel and surgery than patients pre-pandemic. This could be due to a reallocation of personnel across wards, since many nurses and practitioners were shifted to the emergency treatment of SARS-CoV-2 patients. On the other hand, economies of scale might account for the reduction of per-capita costs in interventions with ventilator support. The increased costs sustained for SARS-CoV-2 negative patients with respect to those hospitalized before the pandemic, mainly due to increased use of diagnostics and PPEs, especially for DRGs unrelated to respiratory infections, suggest that the economic burden on the health care system during the pandemic was higher than that just due COVID-19 cases. This should be accounted for by policy makers in future health technology assessment during periods of healthcare strain, as suggested by Brassel et al.^17^.

One limitation of our study is that we could not account for possible changes in admission patterns other than age, sex, type of admission, use of surgery, ICU or CCU, during the pandemic. These could indeed impact the increased costs estimated for patients discharged during the pandemic and be one potential explanation for the increased costs observed even for SARS-CoV-2 negative patients, especially if admission patterns were driven by the severity of cases. In addition, we should note that the estimate of the absorbed human resources costs does not consider the opportunity costs due to the usage of health professionals temporarily taken from other departments to cover the COVID-19 wards shifts. At its peak, in 2020, this phenomenon counted more than 100 health workers on sick leave at the same time. Sample size in each DRG group is not enough to assess the presence of temporal trends over the four year time period among positive patients, however an additional analysis performed on the overall sample of negative patients considering the semester of discharge as a covariate reveals there is no evidence of such effect, except for an upward shock corresponding with the beginning of the pandemic. For positive patients, we acknowledge that potential learning effects in the management of COVID-19 disease, and changes in either patterns of admissions or severity of cases, could lead to decreasing costs over time but our sample did not allow us to explore these effects.

While a previous study analyzed hospitalizations during the pandemic in Italy to quantify the costs related to the hospital management of COVID-19 positive patients in 2020^12^, the strength of our work relies on the comparison of costs between patients discharged with the same DRG before and during the pandemic, and between patients discharged during the pandemic with or without a diagnosis of Covid-19.

Our analysis was performed with data from a large for-profit Research Hospital in the Milan area accredited with the NHS. The ownership is with a large industrial group with a recognized strong focus on efficiency, lean management and logistics teams to support the clinical work. The provider has been accredited by The Joint Commission, a quality accreditation organization for hospitals which conducts onsite reviews at three-year intervals, since 2002. As part of the renewal of this accreditation, the staff performs training, including simulations in preparation for disease outbreaks due to respiratory viruses. In February 2020 a dedicated strategic team was established to keep regular contact with other countries (i.e., China in the early days of the pandemic) and participate in regional and national emergency networks. On the other side, disruption on the supply side (e.g., shortages of PPE, ventilators…), especially in the first phases of the pandemic, were as severe as in other hospitals involved in the care of COVID-19 patients. All together, these elements suggest our estimates of hospital incremental costs due to COVID-19 pandemic and SARS-CoV-2 infection are conservative. Other hospitals in Italy, especially public hospitals, have likely incurred higher costs than those observed in this study. Indeed, a summary published by the Bussola project^18^ with costs related to COVID-19 hospitalizations from 62 hospitals, although following a different costing methods than ours, concluded a 19% efficiency loss was reported with a total economic loss of 119% compared to the DRG tariffs in 2020.

Nonetheless, we believe our study represents a valuable contribution to existing literature as it provides additional evidence, based on individual patient data, for the incremental costs and pressure exerted by the pandemic emergence on the Italian hospital system, from both the extensive (i.e., the number of patients) and the intensive (i.e., the cost of each hospitalization) margins. For policy makers and hospital managers, who had and are currently dealing with the economic burden posed by pandemic, these results contribute to the managerial debate around the transparent definition of tariffs for hospital admissions and, overall, to the ongoing policy debate around the sustainability of the Italian healthcare system.

## Methods

We conducted a repeated cross-sectional analysis on hospitalization episodes occurred in a large research hospital in Milan, Lombardy (Humanitas Research Hospital). In order to cover sufficiently long time period before and after the pandemic outbreak, we analyze discharges occurred between 1st January, 2018 and 31st December 2021.

The Independent ethical committee of IRCCS Humanitas Research Hospital reviewed and approved this study. They confirmed the analyses on retrospective data herein conducted were in line with the informed consent regularly obtained by patients. All methods were carried out in accordance with relevant guidelines and regulations.

Humanitas Research hospital is a highly specialized clinical, research and teaching hospital located south of Milan. It is a private for-profit hospital accredited by the Italian National Health Care System. The hospital works with 759 beds, 38 operating rooms and manages more than 45.000 inpatient admissions, around 57.000 Emergency department accesses and more than 2 million outpatients on an annual basis.

In the period between March 2020 and December 2021 the hospital treated as inpatient 2.986 patients positive for SARS-CoV-2 through 4 waves of disease. The peak of beds needed to treat the patients has been 270 ordinary and 40 intensive care beds.

Among all hospitalizations we selected those associated with the following Diagnosis Related Groups (DRGs): respiratory infections and inflammations with (079) or without (080) complication or comorbidity (CC), simple pneumonia and pleurisy with (089) and without (090) CC, pulmonary edema and respiratory failure (087), respiratory system diagnosis with ventilator support >  = 96 (565) or < 96 h (566), Intracranial Hemorrhage or Cerebral Infarction (014) and kidney and urinary tract infections with CC (320).

Costing is time and effort consuming and, depending on the objective of the analysis, researchers need to decide how accurate and precise cost estimates need to be. In this study we followed a costing approach that can be classified as top-down micro-costing^19^. Micro-costing due to the significant degree of disaggregation used in the identification and measurement of resource and cost components, top-down due to the method for the valuation of resource and cost components that relied on Humanitas Research Hospital detailed internal accounting system. Resources used during the inpatient episodes are routinely collected by the hospital data warehouse where all clinical and administrative events are gathered: diagnostic exams, blood transfused, drugs administered, implants. The monetary value of these resources is estimated using the internal actual cost reported by the hospital. Activity-based costing has been used to account for surgery/operating room usage, ICU stay, and CCU stay. Time was the driver chosen to apportion personnel and PPEs use. Clinical time (spent by physicians, nurses and healthcare professionals) per day of stay for patients with a positive test for SARS-CoV-2 was on average 25% longer than an ordinary inpatient episode according to the specific organization of the shifts. The monetary value is estimated using the average hospital cost per type of employee per day of patient stay. The estimation of unit costs took into consideration the specific bonuses and incentives guaranteed by the hospital during the pandemic. In March 2020, specific cost centers were created to precisely determine the consumption in terms of PPE (Personal Protection Equipment) and other materials connected with the COVID-19 patient care. These costs are added to hospitalization episodes according to the length of stay in hospital.

Data on age, sex and type of hospitalization (emergency room or elective) were recorded at hospital admission, while data on the total length of hospital stay, duration of surgery, intensive care admission (ICU) and length of ICU stay, coronary care unit (UCC) admission and length of UCC stay, unit and date of discharge, and the DRG code were recorded at hospital discharge. If hospitalized patients tested positive for SARS-CoV-2 either at admission or during their stay a flag indicating whether the patient was positive for SARS-CoV-2 was recorded.

The primary outcome of our analysis is the full hospital costs of each hospitalization, computed across different cost items: personnel (including physicians, nurses and health care workers), diagnostics, drugs and treatments, implants, blood transfusion and all other consumables (i.e. medical gases, PPEs, etc.), operating room, ICU and CCU time.

We compared these costs across different DRG-homogenous groups of patients based on their discharge date and SARS-CoV-2 status both in univariate and multivariate analyses. More specifically, we defined three different types of hospitalization: inpatients discharged before 21st February 2020, i.e. before the first SARS-CoV-2 case was notified by Italian Health authorities (reference group), inpatients discharged after this date with a negative SARS-CoV-2 test, and inpatients discharged after this date with at least one positive SARS-CoV-2 test during the hospital admission.

Firstly, costs associated with different DRG codes were compared across the three groups using a stochastic dominance test. Graphical inspection of empirical distribution functions and a one-sided Kolmogorov–Smirnov (KS) test were adopted to assess the stochastic dominance of the distribution function of the hospitalization costs for patients discharged before the pandemic and for those discharged after without a SARS-CoV-2 diagnosis over that of SARS-CoV-2 positive patients.

To better disentangle the association between sustained costs and the different groups of patients defined by their discharge date and SARS-CoV-2 status, we run generalized linear models on the total costs considering the type of patient as the exposure and adjusting for relevant available covariates.

In particular, we modeled the outcome through generalized linear model assuming costs are distributed according to a Gamma distribution and using a logarithmic link function.

Let **Y** be our response variable and **X** the matrix with the considered covariates, the model can be written as

$$ {\text{E}}\left( {{\mathbf{Y}}|{\mathbf{X}}} \right) = {\text{exp}}({\mathbf{\upbeta }}*{\mathbf{X}}) $$

with **Y** ~ Gamma.

Running the model on all the observed hospitalizations allowed to compare costs before and after the pandemic, differentiating between patients with and without SARS-Cov2. We also adjusted for a series of covariates, i.e. age of the patients, sex of patient, the type of admission (elective vs. emergency), the type of discharge (death, transfer to another hospital vs. other), the total length of stay in hospital and three dummy variables indicating whether the hospitalization required surgery, intensive care and UCC admission. A quadratic and cubic terms for the length of stay in hospital were introduced due to the non-linear relation of this variable with the total costs. Following Jones et al.^20^, we also performed additional approaches in modelling the distribution of healthcare costs to check the robustness of our results. Statistical analyses were performed using R version 3.6.2.

### Supplementary Information

Supplementary Information.

## Data Availability

The data that support the findings of this study are available from Humanitas Research Hospital but restrictions apply to the availability of these data, which were used under license for the current study, and so are not publicly available. Data are however available from the authors upon reasonable request and with permission of Humanitas Research Hospital.
