# Author Correction: A repeated cross-sectional analysis on the economic impact of SARS-CoV-2 pandemic at the hospital level in Italy

**DOI:** 10.1038/s41598-023-41716-y

**Published:** 2023-09-05

**Authors:** Filippo Trentini, Oriana Ciani, Elena Vanni, Simone Ghislandi, Aleksandra Torbica, Elena Azzolini, Alessia Melegaro

**Affiliations:** 1 Dondena Centre for Research on Social Dynamics and Public Policy, Bocconi University, 1 Roentgen St., Milan, Italy; 2 Covid Crisis Lab, Bocconi University, Milan, Italy; 3 Center for Health Emergencies, Bruno Kessler Foundation, Trento, Italy; 4 Centre for Research on Health and Social Care Management (CERGAS), SDA Bocconi School of Management, Milan, Italy; 5 IRCCS Humanitas Research Hospital, Milan, Italy; 6 Department of Social and Political Sciences, Bocconi University, Milan, Italy; 7 Department of Biomedical Sciences, Humanitas University, Milan, Italy

Correction to: *Scientific Reports* 10.1038/s41598-023-39592-7, published online 31 July 2023

The original version of this Article contained errors in the name of authors Filippo Trentini, Oriana Ciani, Elena Vanni, Simone Ghislandi, Aleksandra Ghislandi, Elena Azzolini and Alessia Melegaro which were incorrectly given as Trentini Filippo, Ciani Oriana, Vanni Elena, Ghislandi Simone, Torbica Aleksandra, Azzolini Elena and Melegaro Alessia.

The original Article has been corrected.

