# Supplementary material for: A repeated cross-sectional analysis on the economic impact of SARS-CoV-2 pandemic at the hospital level in Italy
Source: Sci Rep. 2023 Jul 31;13:12386. doi: 10.1038/s41598-023-39592-7 (PMC10390582; doi:10.1038/s41598-023-39592-7)
Supplement: Supplementary file 1 — Supplementary Information. [file 41598_2023_39592_MOESM1_ESM.docx]

**A repeated cross-sectional analysis on the economic impact of SARS-CoV-2 pandemic at the hospital level in Italy**

**Appendix**

**Table S1 (appendix). Median sustained episode costs (in EUR) by type of resource for patients with different DRGs by hospital discharge period and SARS-CoV-2 diagnosis**

| **GROUP** | **DRG CODE** | **PERSONNEL** | **SURGERY** | **INTENSIVE CARE** | **UCC** | **DIAGNOSTICS** | **DRUGS** | **IMPLANTS** | **BLOOD TRANFUSION** | **PPEs AND MEDICAL GASES** | **TOTAL** | **Incremental TOTAL COST Pandemic and SARS-CoV-2 negative vs Pre-pandemic** | **Incremental TOTAL COST Pandemic and SARS-CoV-2 positive vs Pre-pandemic** |
| --- | --- | --- | --- | --- | --- | --- | --- | --- | --- | --- | --- | --- | --- |
| **Pre-pandemic** | 14 | 1183.7 (902.6 -1745.8) | 271.6 (210.2 -345.6) | - | 32.2 (25.7 -38.7) | 922.7 (725.5 -1272.6) | 12.5 (4.2 -48) | 1765 (1205 -3546.8) | 362 (181 -422) | - | 2257.3 (1703.5 -3314.1) | - | - |
| **Pandemic and SARS-CoV-2 negative** | 14 | 1043.2 (762.1 -1605.2) | 236.4 (210.2 -329.2) | - | - | 1106.2 (831.6 -1513.2) | 16.4 (5 -64) | 1120 (780 -2591.9) | 362 (169.8 -402.2) | 119.2 (85.2 -171.7) | 2386.2 (1852.7 -3526.1) | 128,9 | - |
| **Pandemic and SARS-CoV-2 positive** | 14 | 1951.7 (1091.6 -4876.1) | 254.5 (254.5 -254.5) | - | 2.7 (1.7 -3.8) | 1508.2 (962.9 -2166.6) | 40 (5.7 -135.3) | - | 362 (271.5 -1801) | 874.1 (582.7 -2258) | 4814.9 (2674.3 -10700.5) | - | 2557,6 |
| **Pre-pandemic** | 79 | 2028­.3 (1242.4 -3108) | 155.3 (154.5 -268.2) | - | 97 (58.8 -144.5) | 1215.2 (685.1 -1961) | 232.3 (94.8 -626.8) | 667.2 (667.2 -667.2) | 362 (181 -543) | - | 3878.7 (2460.6 -6193.7) | - | - |
| **Pandemic and SARS-CoV-2 negative** | 79 | 1710.8 (1202.7 -2726.9) | 178 (128.8 -198.9) | - | 18.9 (18.9 -18.9) | 1508 (963.2 -2297.9) | 208.3 (92.3 -503.2) | - | 563 (362 -1136.2) | 204.4 (140.5 -334.2) | 3870.9 (2603 -6212.1) | -7,8 | - |
| **Pandemic and SARS-CoV-2 positive** | 79 | 1745.5 (1091.6 -2665.1) | 275 (165.9 -525) | - | - | 857.4 (636.4 -1225.3) | 34.3 (15.9 -98.1) | 750 (750 -750) | 362 (181 -563) | 874.1 (509.9 -1384) | 3495 (2324 -5643.2) | - | -383,7 |
| **Pre-pandemic** | 80 | 1393.2 (821.6 -2003.3) | 111.7 (107 -116.5) | - | - | 788 (450.3 -1358.2) | 81.3 (29.2 -247) | - | 181 (181 -316.8) | - | 2650.3 (1456.8 -3709.2) | - | - |
| **Pandemic and SARS-CoV-2 negative** | 80 | 1202.7 (948.7 -1806) | 132.6 (132.6 -132.6) | - | - | 974.5 (689.9 -1614.1) | 85.6 (39 -237.2) | - | 1072 (659 -1448) | 140.5 (111.8 -215) | 2716.7 (2036.6 -3900.8) | 66,4 | - |
| **Pandemic and SARS-CoV-2 positive** | 80 | 1199.7 (732.1 -1579) | - | - | - | 670.3 (542.3 -864.4) | 14.8 (7.1 -32.8) | - | 362 (362 -362) | 603.7 (402.4 -905.5) | 2516.9 (1786.5 -3244) | - | -133,4 |
| **Pre-pandemic** | 87 | 1329.7 (694.6 -2091.8) | 190.5 (175 -285.2) | - | 51.5 (48.8 -113.4) | 713.6 (473 -1102.2) | 80.8 (37.2 -165.3) | 370 (370 -370) | 241.4 (161.6 -442.4) | - | 2433.4 (1406.6 -3887.1) | - | - |
| **Pandemic and SARS-CoV-2 negative** | 87 | 1329.7 (948.7 -2091.8) | 182.2 (172.9 -191.5) | - | - | 887.4 (588.2 -1382.7) | 77.1 (31.9 -160.3) | - | 362 (201.4 -643) | 163.9 (104 -270.7) | 2707.4 (1863.3 -4046.5) | 274 | - |
| **Pandemic and SARS-CoV-2 positive** | 87 | 2665.1 (1745.5 -3738) | 250 (236.4 -390.2) | - | - | 1140.3 (824.9 -1590.2) | 100.1 (49.8 -235.7) | 750 (750 -750) | 362 (362 -497.8) | 1311.1 (905.5 -1911.6) | 5350.9 (3735.6 -7697.5) | - | 2917,5 |
| **Pre-pandemic** | 89 | 1202.7 (821.6 -1964.8) | 166.7 (123.1 -207.4) | - | 11.5 (11.5 -11.5) | 755.6 (489.2 -1187.2) | 97.9 (41 -216.3) | 750 (750 -1195) | 362 (201 -611.1) | - | 2331 (1482.8 -3536.8) | - | - |
| **Pandemic and SARS-CoV-2 negative** | 89 | 1329.7 (948.7 -1964.8) | 153.4 (121.2 -194.3) | - | - | 1014.8 (694.3 -1604) | 89.9 (40.9 -178.4) | 467.5 (326.2 -608.8) | 442 (362 -1303.5) | 153.3 (109.3 -234.2) | 2680.4 (1915.3 -3992.6) | 349,4 | - |
| **Pandemic and SARS-CoV-2 positive** | 89 | 1592.2 (1112 -2489.8) | 241.3 (154.4 -345.5) | - | 44.7 (44.7 -44.7) | 869.3 (629.6 -1331.1) | 77 (40.6 -157.4) | 750 (750 -750) | 362 (186 -402) | 1006.1 (603.7 -1509.2) | 3800.3 (2444.2 -5582.3) | - | 1469,3 |
| **Pre-pandemic** | 90 | 1202.7 (821.6 -1456.7) | 174.2 (132.6 -286.4) | - | - | 688.2 (459.9 -1072.2) | 72.9 (30.2 -146.7) | 375 (300 -450) | 402 (362 -925) | - | 2018.8 (1400.9 -2663.5) | - | - |
| **Pandemic and SARS-CoV-2 negative** | 90 | 948.7 (639.6 -1329.7) | - | - | - | 978 (599.8 -1472.6) | 44.9 (11.2 -96.3) | - | 362 (362 -402) | 109.3 (68.1 -153.3) | 2297.1 (1590.3 -2930.7) | 278,3 | - |
| **Pandemic and SARS-CoV-2 positive** | 90 | 1285.7 (979.1 -1535.7) | 155.3 (155.3 -155.3) | - | - | 574.8 (490.2 -741.9) | 53.7 (20.2 -68.5) | - | 362 (362 -452.5) | 704.3 (578.5 -905.5) | 2639.8 (2074.7 -3137.6) | - | 621,0 |
| **Pre-pandemic** | 320 | 1202.7 (694.6 -1811) | 286.4 (231.8 -362.9) | - | - | 691.8 (446.2 -1004) | 73.9 (34 -173.4) | - | 362 (181 -492.5) | - | 2098.5 (1382.8 -2977.2) | - | - |
| **Pandemic and SARS-CoV-2 negative** | 320 | 1202.7 (821.6 -1964.8) | 247.3 (202.7 -289.8) | - | - | 884.7 (665 -1243.9) | 83 (45.3 -160.9) | - | 362 (181 -470) | 140.5 (93.7 -234.2) | 2395.5 (1780 -3663.7) | 297 | - |
| **Pandemic and SARS-CoV-2 positive** | 320 | 1898.8 (1209 -4121.2) | 340.9 (340.9 -340.9) | - | - | 1471.8 (877.1 -1886) | 130.8 (62.5 -334.6) | - | 543 (543 -543) | 1207.3 (629.6 -1930.3) | 4651.9 (2975.8 -7867.5) | - | 2553,4 |
| **Pre-pandemic** | 565 | 1837.8 (567.6 -2472.9) | 253.4 (223.3 -283.5) | 16244.8 (10621.6 -19993.6) | 7.2 (6.1 -21.4) | 2589.2 (1653 -3357.6) | 106.2 (55.6 -299.8) | 1652 (1652 -1652) | 543 (342.9 -975.4) | - | 22547.8 (15962.3 -26863.2) | - | - |
| **Pandemic and SARS-CoV-2 negative** | 565 | 821.6 (254 -2345.9) | - | 14995.2 (9996.8 -17494.4) | - | 2767.6 (2249.4 -5128.9) | 119.2 (37.7 -196.3) | - | 382 (181 -688.8) | 272.5 (195.1 -525.1) | 20349 (13545.4 -26434.8) | -2198,8 | - |
| **Pandemic and SARS-CoV-2 positive** | 565 | 2971.7 (1526.2 -4810.9) | 186.4 (186.4 -186.4) | 0 (0 -14995.2) | 124.8 (72 -158.6) | 2435.1 (1635.9 -3372.8) | 81 (33.9 -140) | 230 (115 -345) | 392 (201 -721.6) | 2314 (1509.2 -3219.5) | 12451.5 (8573.8 -20783.5) | - | -10096,3 |
| **Pre-pandemic** | 566 | 1075.7 (194.5 -1710.8) | 345.5 (345.5 -345.5) | 4998.4 (1249.6 -7497.6) | 9.1 (8.8 -9.4) | 1391.4 (1022.5 -2067.9) | 56 (34.1 -311.9) | - | 241.4 (201 -656.8) | - | 7532.3 (3745.2 -11340.9) | - | - |
| **Pandemic and SARS-CoV-2 negative** | 566 | 294.5 (194.5 -916.9) | - | 2499.2 (1249.6 -3748.8) | - | 1453.3 (911.2 -2198.6) | 94.9 (19 -261.2) | - | 243 (242.2 -1757.9) | 57.5 (31.2 -174.6) | 4621.9 (3181.3 -7102.2) | -2910,4 | - |
| **Pandemic and SARS-CoV-2 positive** | 566 | 688.1 (478.6 -1439) | 336.4 (336.4 -336.4) | 0 (0 -624.8) | 14.8 (14.8 -14.8) | 835.9 (569.6 -1192.7) | 19.5 (13.8 -58.2) | - | 362 (241.4 -362) | 301.8 (218.5 -801.2) | 2299.7 (1528.2 -5135) | - | -5232,6 |

**Table S2 (appendix). Mean estimates of the increases in costs among different groups of patients (reference: patients discharged before the pandemic) obtained with the generalized linear model and with the log-linear model.**

|  | **COMPARISON** | **DRG** | **DRG DESCRIPTION** | **DELTA COSTS**  **mean (95%CI)** | **PVALUE** | **AIC** | **DELTA COSTS**  **mean (95%CI)** | **PVALUE** | **Adjusted R2** | **DIFFERENCE** | **SAMPLE SIZE**  **(pre-pandemic; pandemic negative; pandemic positive)** |
| --- | --- | --- | --- | --- | --- | --- | --- | --- | --- | --- | --- |
| **RESPIRATORY** | Pandemic negative vs prepandemic | 79 | Respiratory infections and inflammations with complication or comorbidity (CC) | 23.59 (18.39;29.02) | 0 | 22005,8 | 22.73 (18.26;27.36) | 0 | 0,91 | 0,86 | N_pre=252; N_post=211; S=897 |
|  | Pandemic positive vs prepandemic | 79 | Respiratory infections and inflammations with complication or comorbidity (CC) | 31.32 (26.95;35.82) | 0 | 22005,8 | 34.79 (30.91;38.78) | 0 | 0,91 | -3,47 | N_pre=252; N_post=211; S=897 |
|  | Pandemic negative vs prepandemic | 87 | pulmonary edema and respiratory failure | 13.13 (7.8;18.76) | 0 | 13043,8 | 12.96 (8.14;17.99) | 0 | 0,93 | 0,17 | N_pre=241; N_post=126; S=441 |
|  | Pandemic positive vs prepandemic | 87 | pulmonary edema and respiratory failure | 47.47 (41.26;53.94) | 0 | 13043,8 | 50.94 (45.2;56.9) | 0 | 0,93 | -3,47 | N_pre=241; N_post=126; S=441 |
|  | Pandemic negative vs prepandemic | 89 | simple pneumonia and pleurisy with complication or comorbidity (CC) | 18.22 (13.95;22.65) | 0 | 25584,1 | 18.94 (15.4;22.58) | 0 | 0,86 | -0,72 | N_pre=647; N_post=519; S=415 |
|  | Pandemic positive vs prepandemic | 89 | simple pneumonia and pleurisy with complication or comorbidity (CC) | 39.22 (33.61;45.08) | 0 | 25584,1 | 45.25 (40.39;50.28) | 0 | 0,86 | -6,03 | N_pre=647; N_post=519; S=415 |
|  | Pandemic negative vs prepandemic | 80 | Respiratory infections and inflammations without complication or comorbidity (CC) | 22.47 (11.82;34.16) | 0 | 4350,42 | 23.14 (13.41;33.7) | 0 | 0,86 | -0,67 | N_pre=60; N_post=50; S=170 |
|  | Pandemic positive vs prepandemic | 80 | Respiratory infections and inflammations without complication or comorbidity (CC) | 35.3 (25.15;46.17) | 0 | 4350,42 | 38.93 (29.58;48.95) | 0 | 0,86 | -3,63 | N_pre=60; N_post=50; S=170 |
|  | Pandemic negative vs prepandemic | 90 | simple pneumonia and pleurisy without complication or comorbidity (CC) | 15.82 (3.76;29.39) | 0,008 | 8902,18 | 19.61 (12.64;27.01) | 0 | 0,73 | -3,79 | N_pre=244; N_post=143; S=168 |
|  | Pandemic positive vs prepandemic | 90 | simple pneumonia and pleurisy without complication or comorbidity (CC) | 28.11 (14.65;43.22) | 0 | 8902,18 | 36.47 (28.33;45.13) | 0 | 0,73 | -8,36 | N_pre=244; N_post=143; S=168 |
|  | Pandemic negative vs prepandemic | 565 | respiratory system diagnosis with ventilator support >=96 | 7.85 (-2.32;19.11) | 0,136 | 3943,72 | 9.73 (-1.15;21.8) | 0,081 | 0,93 | -1,88 | N_pre=31; N_post=23; S=157 |
|  | Pandemic positive vs prepandemic | 565 | respiratory system diagnosis with ventilator support >=96 | 25.14 (15.45;35.56) | 0 | 3943,72 | 27.19 (16.89;38.39) | 0 | 0,93 | -2,05 | N_pre=31; N_post=23; S=157 |
|  | Pandemic negative vs prepandemic | 566 | respiratory system diagnosis with ventilator support <96 | -5.14 (-24.17;19.14) | 0,649 | 1829,61 | -4.43 (-23.46;19.33) | 0,686 | 0,87 | -0,71 | N_pre=41; N_post=16; S=47 |
|  | Pandemic positive vs prepandemic | 566 | respiratory system diagnosis with ventilator support <96 | -2.2 (-21.54;22.19) | 0,84 | 1829,61 | 3.79 (-15.85;28.01) | 0,725 | 0,87 | -5,99 | N_pre=41; N_post=16; S=47 |
| **NON RESPIRATORY** | Pandemic negative vs prepandemic | 14 | Intracranial Hemorrhage Or Cerebral Infarction | 17.38 (13.68;21.2) | 0 | 16251,8 | 17.78 (14.9;20.74) | 0 | 0,89 | -0,4 | N_pre=497; N_post=523; S=33 |
|  | Pandemic positive vs prepandemic | 14 | Intracranial Hemorrhage Or Cerebral Infarction | 53.33 (39.87;68.46) | 0 | 16251,8 | 54.67 (43.91;66.24) | 0 | 0,89 | -1,34 | N_pre=497; N_post=523; S=33 |
|  | Pandemic negative vs prepandemic | 320 | kidney and urinary tract infections with complication or comorbidity (CC) | 12.27 (6.97;17.81) | 0 | 6890,37 | 12.79 (8.07;17.72) | 0 | 0,9 | -0,52 | N_pre=178; N_post=247; S=19 |
|  | Pandemic positive vs prepandemic | 320 | kidney and urinary tract infections with complication or comorbidity (CC) | 59.71 (42.03;80.16) | 0 | 6890,37 | 60.99 (44.82;78.96) | 0 | 0,9 | -1,28 | N_pre=178; N_post=247; S=19 |

**FigS1. Empirical cumulative distribution functions of hospitalization costs.** Cumulative distribution of sustained cost for patients hospitalized for intracranial hemorrhage or cerebral infarction (014) (**A**), respiratory infections and inflammations with (079) (**B**) or without (080) (**C**) CC, pulmonary edema and respiratory failure (087) (**D**), simple pneumonia and pleurisy with (089) (**E**) and without (090) (**F**) CC, kidney and urinary tract infections with CC (320) (**G**), respiratory system diagnosis with ventilator support >=96 (565) (**H**) or <96 hours (566) (**I**), discharged in the pre-pandemic period (yellow), during the pandemic without a diagnosis for SARS-COV-2 (blue), and during the pandemic with a diagnosis for SARS-COV-2 (red).

To account for a potential time trend, we run an additional analysis on negative patients discharged with any of the 9 DGRs considered in the main analysis before or during the pandemic and we defined 8 groups of patients based on their time of discharge: 1st and 2nd semester of 2018 (T1 and T2), 1st semester of 2019 (T3), 2nd semester of 2019 up to February 20th 2020 (T4), February 21st 2020 up to July 1st 2020 (T5), 2nd semester of 2020 (T6), 1st and 2nd semester of 2021 (T7 and T8) .

We therefore modeled the outcome through generalized linear model assuming costs are distributed according to a Gamma distribution and using a logarithmic link function.

As shown in FigS2, no such effect was revealed except for an upward shock corresponding with the beginning of the pandemic.

** FigS2. Average marginal effects of time of discharge on the overall cost of hospitalizations estimated by the generalized linear model.**

**
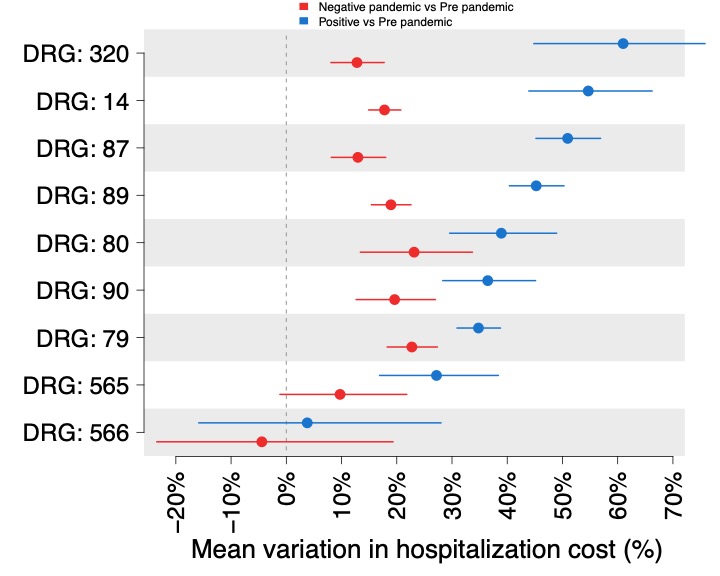
FigS3. Results of the log linear model on the total costs.**

**FigS4. Residual plot for the generalized linear models**

**FigS5. Residual plot for the log-linear models**
